# A MATLAB-based app to improve LC–MS/MS data analysis for N-linked glycan peak identification

**DOI:** 10.1186/s12859-023-05346-5

**Published:** 2023-06-17

**Authors:** Ashna Dhingra, Zayla Schaeffer, Natalia I. Majewska Nepomuceno, Jennifer Au, Joomi Ahn

**Affiliations:** 1 Bioprocess Technologies and Engineering, BioPharmaceuticals R&D, AstraZeneca, Gaithersburg, MD USA; 2 Department of Chemical and Biomolecular Engineering, Whiting School of Engineering, Johns Hopkins University, Baltimore, MD USA; 3 Cell Culture and Fermentation Sciences, BioPharmaceuticals R&D, AstraZeneca, Gaithersburg, MD USA; 4 Analytical Sciences, BioPharmaceuticals R&D, AstraZeneca, Gaithersburg, MD USA

**Keywords:** Glycosylation, Glycans, Tandem mass spectrometry, Matrix-assisted laser desorption/ionization, MATLAB, Liquid chromatography

## Abstract

**Background:**

Glycosylation is an important modification to proteins that plays a significant role in biological processes. Glycan structures are characterized by liquid chromatography (LC) combined with mass spectrometry (MS), but data interpretation of LC/MS and MS/MS data can be time-consuming and arduous when analyzed manually. Most of glycan analysis requires dedicated glycobioinformatics tools to process MS data, identify glycan structure, and display the results. However, software tools currently available are either too costly or heavily focused on academic applications, limiting their use within the biopharmaceutical industry for implementing the standardized LC/MS glycan analysis in high-throughput manner. Additionally, few tools provide the capability to generate report-ready annotated MS/MS glycan spectra.

**Results:**

Here, we present a MATLAB-based app, GlyKAn AZ, which can automate data processing, glycan identification, and customizable result displays in a streamlined workflow. MS1 and MS2 mass search algorithms along with glycan databases were developed to confirm the fluorescent labeled N-linked glycan species based on accurate mass. A user-friendly graphical user interface (GUI) streamlines the data analysis process, making it easy to implement the software tool in biopharmaceutical analytical laboratories. The databases provided with the app can be expanded through the Fragment Generator functionality which automatically identifies fragmentation patterns for new glycans. The GlyKAn AZ app can automatically annotate the MS/MS spectra, yet this data display feature remains flexible and customizable by users, saving analysts’ time in generating individual report-ready spectra figures. This app accepts both OrbiTrap and matrix-assisted laser desorption/ionization–time of flight (MALDI–TOF) MS data and was successfully validated by identifying all glycan species that were previously identified manually.

**Conclusions:**

The GlyKAn AZ app was developed to expedite glycan analysis while maintaining a high level of accuracy in positive identifications. The app’s customizable user inputs, polished figures and tables, and unique calculated outputs set it apart from similar software and greatly improve the current manual analysis workflow. Overall, this app serves as a tool for streamlining glycan identification for both academic and industrial needs.

**Supplementary Information:**

The online version contains supplementary material available at 10.1186/s12859-023-05346-5.

## Background

Glycosylation is a posttranslational modification involving the addition of glycans, chain-like branches of covalently linked monosaccharides, to a protein during synthesis [1]. The glycosylation profile of a therapeutic protein can impact its half-life, efficacy, and interactions with the human body [1]. As a result, characterizing and maintaining a consistent glycosylation profile in a therapeutic protein is crucial, especially for regulatory approval and quality assurance. Characterization of glycan species is made difficult by the abundance and diversity of different glycans, many of which are structural isomers, as well as the range of glycan sites that may be available on a glycoprotein.

Glycan species can be separated into N-linked and O-linked glycans. These glycans differ in how they attach to the glycoprotein, as well as in their base structures. N-Linked glycans link to asparagine residues in the pattern Asn-X-Ser/Thr, where X is any amino acid but proline, whereas O-linked glycans can link to any serine or threonine present on the glycoprotein [2]. N-Linked glycans also have a common core structure of two N-acetylglucosamine units attached to three mannose units, two of which branch out from the first. O-Linked glycans, on the other hand, have no common core structure [2] and are out of the scope of this work.

A number of approaches can be used to analyze glycoproteins by mass spectrometry (MS). Glycoproteins can be analyzed as intact structures, digested into glycopeptides, or released as intact glycans with fluorescently labeled tags [3]. In the last case, the glycan is removed from the protein by enzymatic release, using peptide-N-glycosidase (PNGase) F or A. A fluorescent tag or label, such as 2-aminobenzoic acid, 2-aminobenzamide (2-AB), or procainamide (ProCA), can react with an aldehyde group at the base of the glycan chain to enable quantification of the glycan by fluorescence [4]. In tandem MS (MS/MS), the tagged glycans are first passed through MS1, where they are ionized by one (commonly H^+^) or multiple charge states and separated by their mass-to-charge ratio (m/z); the abundance of glycans at each m/z is quantified by the fluorescence tag intensity of each glycan species. After the first pass through the analyzer, the tagged glycans are directed to a collision cell and fragmented. Several ion dissociation techniques are available for MS/MS analysis, such as collision-induced dissociation (CID), higher-energy collisional dissociation (HCD), electron transfer dissociation (ETD), and electron capture dissociation (ECD), each favoring a specific bond breakage resulting in a dominant ion type [3]. For example, CID, in which the glycans collide with inert gas molecules, usually results in B- and Y-ions [3]. Other types of ions include A- and X-ions, which are cross-ring cleavages caused by HCD [5], and C- and Z-ions, which are formed through ETD and are used to determine the site of glycosylation on the peptide backbone [6]. This work focuses only on B- and Y-ion fragmentation, which is the most informative for determining glycan structure.

Currently used analyst-based characterization workflows can be labor intensive and time consuming, providing an opportunity to improve the process of characterization. When reviewing mass spectra of intact glycans, analysts may have to manually match m/z and retention time (RT) peaks to custom glycan databases. For MS/MS, analysts may need to manually determine potential fragmentation patterns for each parent glycan and cross-check their presence in MS/MS spectra. They might then need to construct figures that annotate and visually show the fragments matched to their corresponding peaks for written reports or regulatory submissions. All of this is both time consuming and repetitive for the analyst.

Both commercial and open-source software are available to assist with analyzing MS/MS data. SimGlycan software, which is available through a yearly subscription, predicts and scores the presence of glycan structures from its database in MS/MS data [7]; however, the cost may be prohibitive for academic and some industry users. Early open-source tools developed to aid glycan and glycopeptide identification include Cartoonist [8], GlycoFragment [9], and GlycoSearchMS [10]. These and similar tools often focus on automating one or a few portions of the spectrum identification workflow, although some are no longer supported. In recent years, more contemporary and novel bioinformatics resources for MS-based glycomics have become available in public domain. These resources include glycan databases such as the NIST 20 Tandem MS Library [11] and GlyTouCan [12]; released glycan identification software such as GlycoWorkbench [13], GlycoMaster DB [14], Glycoforest 1.0 [15], GlycoNovoDB [3], GRITS Toolbox [16]; and glycopeptide identification software such as GlycReSoft [17], GlycoSpectrumScan [18], and pGlyco3 [19]. These resources focus on increasing or augmenting automation of the MS peak identification workflow, broadening the scope of use cases, and improving the integration of glycan databases and statistics. Some of these tools, however, have a high level of user complexity due to the variety of use cases available, making them difficult to navigate. In addition, these tools can be fast to automate data analyses but show a lack of user-friendly customizable features to generate report-ready figures in MS and MS/MS glycan spectra as the analyses outputs, making it difficult to implement in high-throughput industry analytical laboratories.

The GlyKAn AZ app was developed to address these challenges, keeping in mind the needs of an industrial analytical setting. It processes LC/MS and MS/MS data acquired by Orbitrap MS instruments for released N-linked glycans labeled with a fluorescent tag. During MS/MS processing (referring to data of fragmented glycans only), B-, Y-, and internal glycan ions are considered when annotating the fragmentation pattern, requiring the CID or HCD fragmentation methods for data generation. The GlyKAn AZ app can also process data generated by matrix-assisted laser desorption/ionization–time of flight (MALDI–TOF) MS instrument for permethylated N-linked glycans, which are similarly removed from the glycoprotein backbone. Simpler user interference is required during the analysis process, making it ideal for high-throughput glycan identification. The clearly defined scope for the GlyKAn AZ app reduces its complexity and makes it straightforward for new users to learn.

The GlyKAn AZ app offers several unique features that set it apart from similar software. Its user-specified settings allow customization of the breadth of the outputs. Use of Microsoft Excel enables the user to easily view and understand both the glycan databases and app outputs. The app produces annotated images of MS/MS fragmentation spectra that are well formatted and clear, requiring minimal user effort to adjust them to a report-ready standard. These images are accompanied by adduct identification, as well as by other values, such as fragment masses unique to the glycan in question, that may assist the user with positively identifying spectra. Isomer glycan identification is built into the MS1 and MS/MS operations, and the incorporation of LC data can significantly reduce processing time and the number of false positives. The app streamlines the database generation process by accepting multiple new glycan structures simultaneously, at which time it generates all possible fragment structures and automatically saves them to the fragment database and image repository files. Overall, the GlyKAn AZ app offers exceptional data display options and is compatible with the hardware and software commonly found in research laboratories.

## Implementation

### Materials and methods

#### Sample preparation

Glycans were prepared for analysis by enzymatic release, followed by labeling with a fluorescent tag, either 2-AB or ProCA. Briefly, the antibody sample was prepared in replicate, starting with digestion by PNGase F (Sigma Aldrich) in 50 mM tris(hydroxymethyl)aminomethane (Tris)–HCl buffer, pH 7.8, at 37 °C for 16 ± 4 h to release N-glycans from the antibody Fc region. The released glycans of each replicate were labeled with either 2-AB or ProCA by reductive amination and then extracted with GlykoClean S-Plus cartridges (Agilent).

#### Liquid chromatography and mass spectrometry

Separation was done using an Acquity UPLC with an Acquity UPLC fluorescence (FLR) detector (Waters, Milford, MA). The samples were run on a ACQUITY UPLC Glycoprotein BEH Amide column (130 Å, 1.7 μm, 2.1 mm × 150 mm) with mobile phase A (50 mM ammonium formate, pH 4.4) and mobile phase B (100% acetonitrile) flowing at 0.5 mL/min at a column temperature of 60 °C. The linear gradient was 23–36% A for 23.5 min, 36–40% A for 23.5 to 36 min, and 40–23% A for 36 to 37 min. The flow rate was 0.5 mL/min between minutes 0–34.5 and 36–37 and 0.4 mL/min between minutes 34.5–36. The fluorescence detector excitation and emission wavelengths were set at 310 and 370 nm, respectively. The UPLC-FLR system along with LC–MS analysis were controlled by MassLynx 4.1 software.

Orbitrap Fusion mass spectrometer with an ESI source (ThermoFischer, Waltham, MA) was coupled with UPLC/FLR to analyze 2-AB or PCA-labeled glycans. MS was controlled by ThermoFischer Scientific Xcalibur software. All spectra were recorded at resolution 120,000, and the m/z range was 500 to 2,000. Spray voltage was 4,000 V and the ion transfer tube temperature was 280 °C in the positive ion mode.

#### MALDI–TOF MS

The protein samples were de-glycosylated by overnight digestion at 37 °C, using PNGase-F (Sigma Aldrich). The released glycans were enriched and purified by HILIC solid-phase extraction. The enriched fraction was then dried and derivatized by a permethylation process that involved incubation with dimethylsulfoxide, NaOH, and CH_3_I at room temperature for 2 h. The permethylated glycans were extracted and purified by a liquid–liquid extraction process, using dichloromethane (to extract hydrophobic permethylated glycans) and water (contaminants). Finally, the samples were dried and resuspended in 50% methanol. Super–2,5-dihydroxybenzoic acid (DHB) matrix (Merck Life Science) was prepared in 50% acetonitrile/water containing 0.1% trifluoroacetic acid. Permethylated N-glycans were mixed with the Super-DHB matrix and spotted on a steel MALDI target plate (Bruker Daltonics). Once dried, the plate was inserted and data were acquired in an Ultraflex TOF/TOF mass spectrometer (Bruker Daltonics) with parameters of positive reflector mode (range, 1200–6000 m/z) and a calibrated profile obtained from peptide calibration mix (Bruker). Data were acquired in triplicate. Fragmentation spectra of each glycan peak were acquired in positive LIFT and CID mode for glycan sequencing and confirmation of the glycan species.

### Workflow

**Fig. 1:**
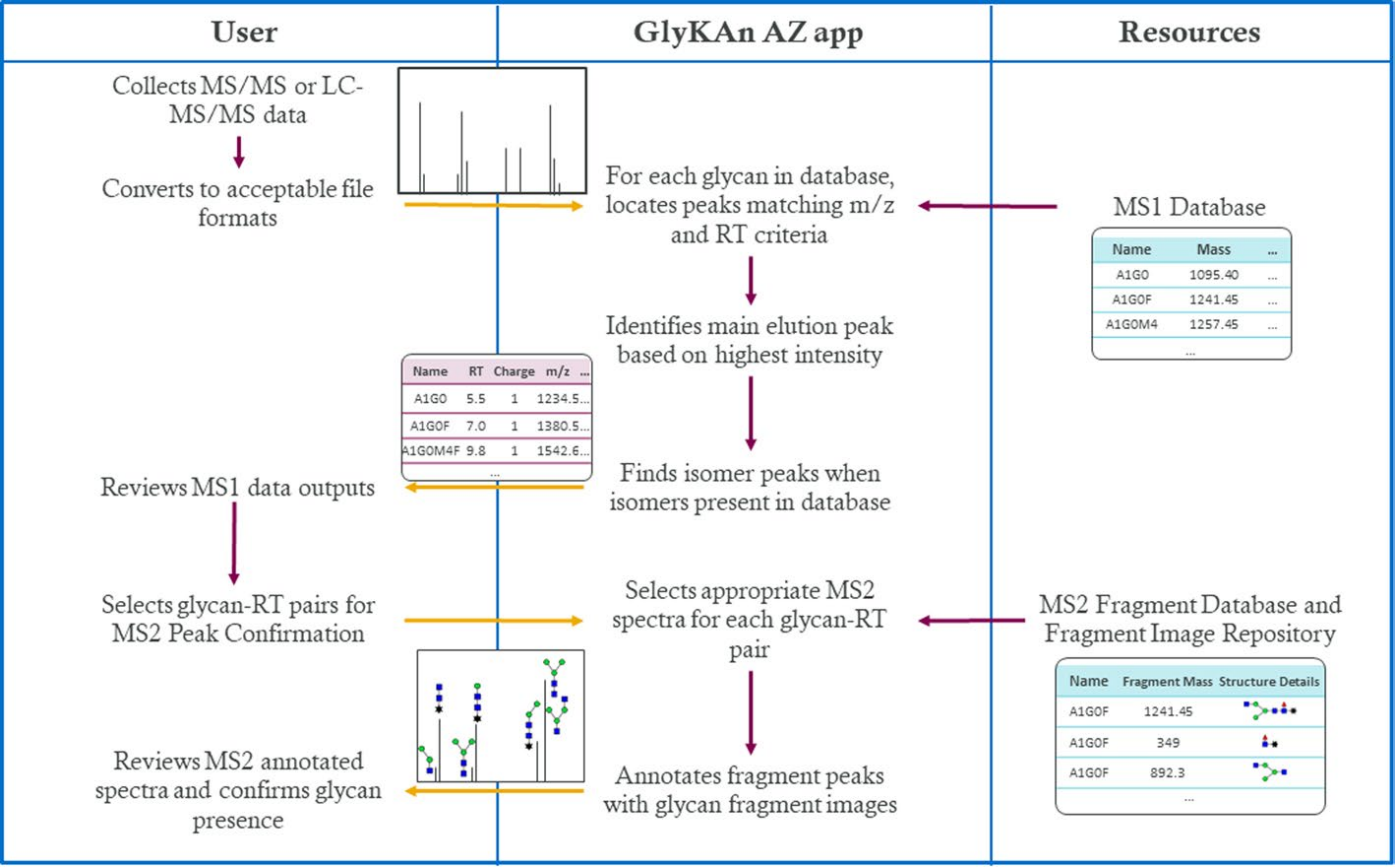
User flow diagram for the GlyKAn AZ app, summarizing the steps an analyst would take to analyze LC–MS/MS data with the app

The GlyKAn AZ app was created to address a need for improving the current MS analysis workflow from manual data interpretation to automated data analysis. The app consists of five steps an analyst would take to analyze MS data with the app (Fig. 1).

1. Convert file formats
2. Identify MS1 peak based on the highest intensity
3. Review MS1 data
4. MS2 peak confirmation
5. MS2 spectra annotation

The app separates analysis of MS/MS data into two separate tabs: MS1, which is an initial screen of masses concerned only with pre-fragmentation glycan data, and MS2, which is a more thorough analysis of post-fragmentation glycan data. The mzXML file and the MS1 Database containing glycan names, theoretical masses (in daltons [Da]), and optionally expected RTs (in minutes) are first loaded into the MS1 tab in the app, and MS1 analysis is performed. The MS1 output Excel file that the app returns can then be reviewed to determine which glycans are likely to be present in the sample. This output file, the MS2 Fragment Database, and the MS2 fragment image repository can then be loaded into the MS2 Peak Confirmation tab. The MS2 Fragment Database lists all possible fragments of the parent glycans present in the MS1 Database and their theoretical masses. The MS2 fragment image repository contains files that pictorially depict the fragments listed in the MS2 Fragment Database. The user selects glycan-RT pairs that will be processed for MS2 analysis. The app then returns annotated and labeled MS2 fragmentation spectra, providing the analyst with a method for glycan confirmation for the selected glycan peaks.

The following sections provide detailed descriptions of the various tabs and information on the algorithms used. A user manual, as well as sample databases for the MS1 and MS2 tabs, are included in the Additional file 1. New glycan structures can be added to the databases using the Fragment Generator tab, which calculates fragmentation masses based on glycan structure.

#### MS1 peak identification

The GlyKAn AZ app is MATLAB R2020b compatible and requires the use of ProteoWizard [20], an open-source tool that converts raw MS data to mzXML format, which is readable by MATLAB [21]. ProteoWizard is used to convert the raw MS files to mzXML format. Optional LC data can be utilized to limit the RT range that the app searches [22]. MassLynx raw data containing LC retention time and fluorescence intensity of glycans can be exported to excel to use it as an optional LC data file.

**Fig. 2:**
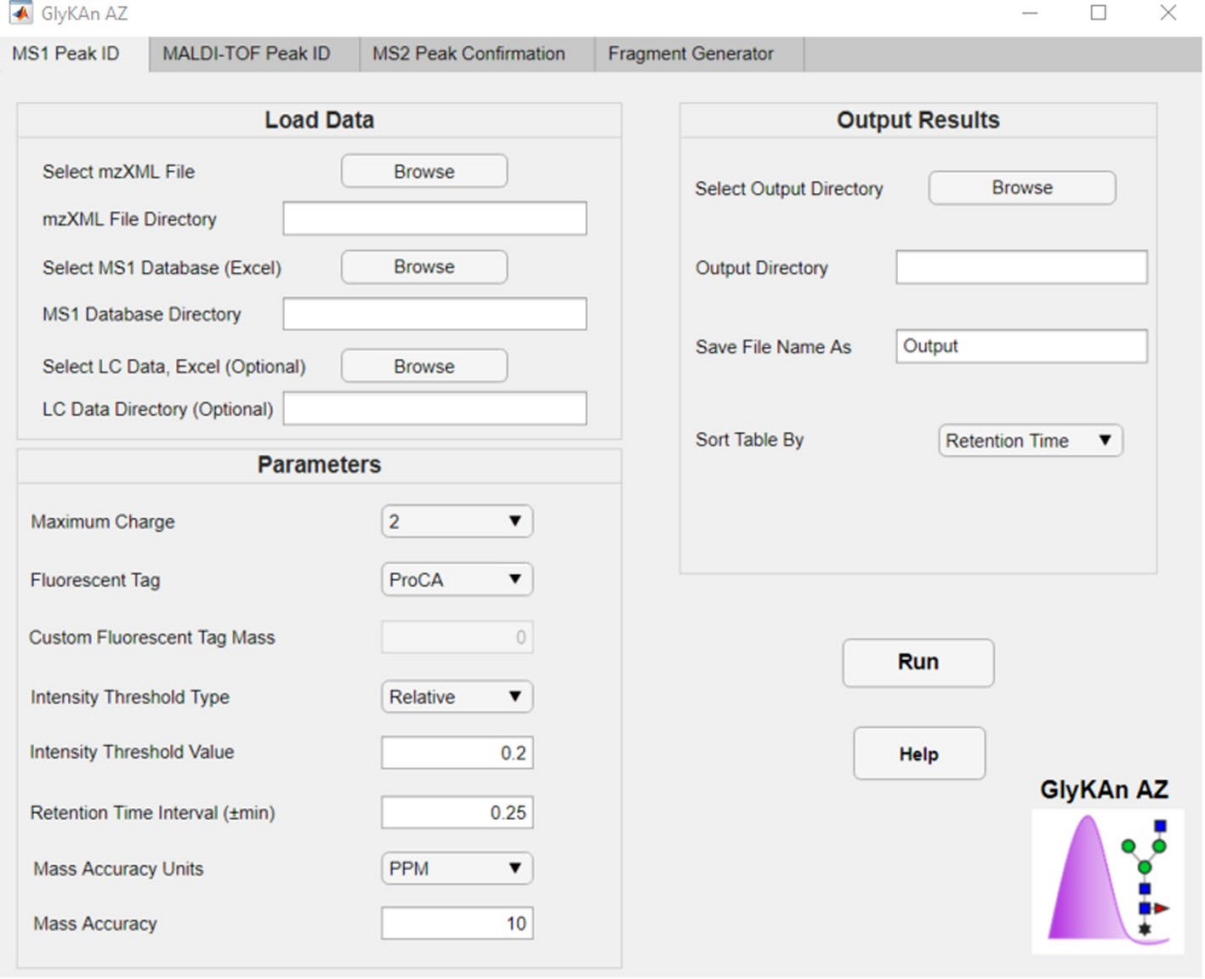
MS1 Peak ID tab of the GlyKAn AZ app

The first tab of the app is the MS1 Peak ID tab, where the user can upload MS data, the MS1 Database, and optional LC data (Fig. 2). The user can edit various default parameters, which are summarized in Table 1. The RT interval setting is applicable only if the third column in the MS1 Database is populated with approximate RTs for each glycan. This field is left blank by default to provide flexibility for the more likely scenario in which the glycans’ approximate RTs are not known in advance. However, once a representative sample is analyzed, the RTs can be included to allow for batch processing of multiple data replicates. “Relative intensity” refers to the intensity of a peak relative to those of other peaks within the same RT scan. Therefore, it is possible for two peaks with the same m/z but different RTs to both have relative intensities of 100%.

**Table 1:** MS1 user-defined settings

User setting	Options	Default value	Available range
Mass accuracy	ppm or Da	10 ppm	> 0
Fluorescent tag	2-AB, ProCA, or custom	ProCA	Custom mass: ≥ 0
Maximum charge states	1, 2, 3, or 4	2	N/A
Intensity threshold type	Absolute or relative (%)	Relative: 25%	Absolute: > 0 Relative: 0–100%
RT interval	Minutes	0.25 min	> 0

**Fig. 3:**
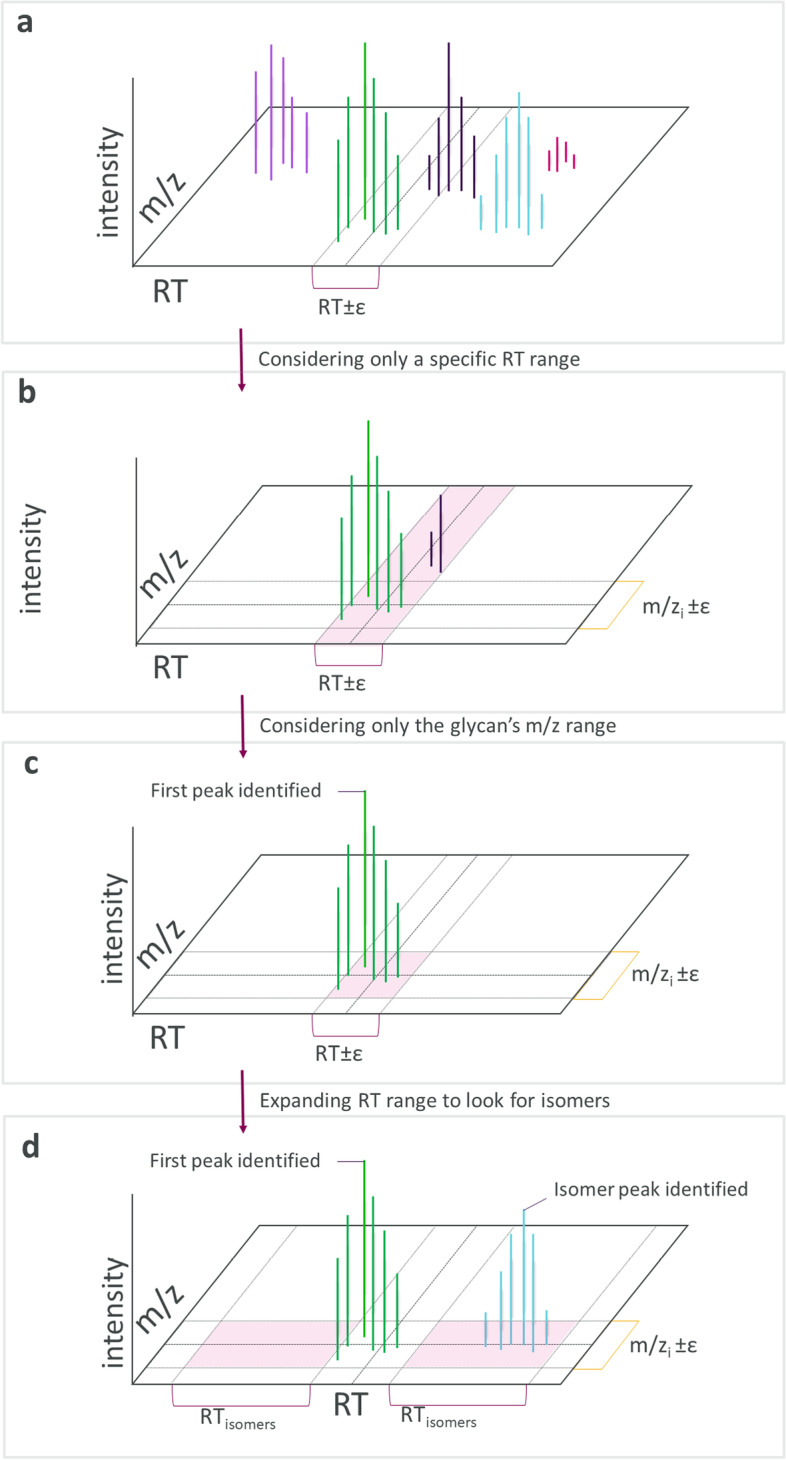
MS1 peak finding. When analyzing MS1 data, the app **a** focuses in on a single RT range if provided in the database, **b** defines the appropriate m/z range for the glycan of interest, **c** identifies the peak of highest intensity as the glycan of interest, and finally, **d** expands the search range to find isomer peaks

A visual representation of the MS1 glycan identification process is shown in Fig. 3. The algorithm for MS1 analysis loops through each glycan in the database and each charge value (where maximum charge value is a user-specified parameter). The app attempts to find the glycan at each charge value in the m/z versus the RT matrix within the user-specified tolerances. When analyzing MS1 data, the app will review all peaks for every glycan in the applicable m/z and RT ranges and select the highest intensity as the first peak. If LC data is provided by the user, then the LC profile has been converted by MassLynx into a series of RT values identifying the start and stop of each elution peak; these LC RT values are converted to MS1 RT values by aligning the highest intensity peak in LC and MS1 spectra. After conversion, these MS1 RT ranges are the only locations where the app will search for glycan matches.

After identifying the highest intensity peak match for a glycan, the app then moves to isomer identification, where the number of isomers that can be identified is equal to the number of glycans in the MS1 Database that share the same mass. Isomer peaks are found at the same m/z within a range of ± 1.75 min around the highest-intensity (first) peak for that m/z. The range is limited because isomer peaks are more likely to elute around the same RT. The app’s algorithm uses the changing slope of the intensity versus RT curve to find the boundaries between peaks. The algorithm does this by generating an intensity versus RT curve for each glycan-m/z pairing. It then calculates the slope between subsequent points. A peak is identified if three of four slope calculations are positive and three of four of the immediately following slope calculations are negative. These criteria ensure that the peak identified is distinct from other peaks of greater intensity.

#### Review MS1 data

The output of the MS1 tab is a Microsoft Excel file that contains the list of glycans identified and the masses, RT, and absolute and relative intensities at which they were identified. This output can then be used by the analyst to determine the likelihood that a glycan is present in the sample, using the absolute and relative intensity values.

If the user chooses to input LC data, the app limits the RT ranges within which it searches to only those ranges where fluorescence peaks occur. The app calibrates the LC time ranges with the MS/MS time ranges by finding the peak with the highest intensity in both spectra.

The MALDI–TOF Peak ID tab operates similarly to the MS1 Peak ID tab. MALDI–TOF data containing m/z and intensity values for non-fragmented glycans, with some formatting modifications, can be loaded into the app along with an Excel database of glycans and their masses. The same user parameters that are available in the MS1 Peak ID tab are also available for the MALDI–TOF Peak ID tab. The output of the MALDI–TOF Peak ID tab is an Excel table similar to the MS1 output table containing the list of glycans identified, the theoretical and observed masses, and the absolute and relative intensities at which they were identified. The analyst can then use these results to positively identify the presence of glycans.

#### MS2 peak confirmation

The MS2 peak confirmation tab of the app can be used once the MS1 Peak ID is completed. The app allows the analyst to select which glycans and corresponding RTs are inputted for MS2 analysis. There is also a “select all” option in the app should the analyst decide to bypass the step of manually curating a glycan list from MS1 results. With the “select all” option, the app will attempt to perform MS2 analysis for all of the glycans that the app identified during MS1 Peak ID. Various settings can be specified as shown in Table 2. The MS2 label relative threshold defines the limit below which spectrum peaks are considered to be noise.

**Table 2:** MS2 user-defined settings

User setting	Options	Default value	Available range
Mass accuracy	ppm or Da	100 ppm	> 0
Fluorescent tag	2-AB, ProCA, or custom	ProCA	Custom mass: ≥ 0 Da
MS2 label relative threshold	N/A	3%	0–100%

The app begins looking for an MS/MS fragment RT at the MS1 RT value minus 0.1 min, and then steps through subsequent MS/MS fragment RTs. The first MS/MS fragment RT identified that matches the m/z and charge value for the precursor mass (parent glycan) is selected for the MS2 spectrum figure. Once the MS2 spectrum is found, the app systematically considers each possible fragment from the MS2 Fragment Database and attempts to identify the fragment in the spectrum. A fragment is identified if the relative intensity exceeds the intensity threshold and the mass accuracy is within the mass accuracy tolerance, both of which are user-specified parameters. The app then annotates each m/z with up to two potential fragments from the proposed parent glycan.

#### MS2 spectra annotation

The outputs of this portion of the app are the annotated fragmentation spectra for each selected parent glycan-RT pair that the app found. There are three tabs in each generated figure. The main tab annotates the MS2 spectra with m/z and charge values indicating matching fragments and fragments with adducts (+ Na, + K, –OH). The Cartoon tab annotates the MS2 spectra with fragment images and m/z values indicating matching fragments. The monosaccharide symbol translation is described in Table 3. The Details tab provides relevant statistics for the analyst to use in determining whether a glycan is present in the sample. The Details tab displays the percentage of glycan fragments identified of the considered possible fragments for that glycan. It also displays the mass of any unique fragments identified that are specific to that glycan versus its isomers.

**Table 3:** Monosaccharide and tag ID code and symbol translation

Monosaccharide	ID Code	Composition	Monoisotopic mass (g/mol)	Symbol
Xylose	Xyl	C_5_H_10_O_5_	150.0528	
Fucose	Fuc	C_6_H_12_O_5_	164.0685	
Alactose	Gal	C_6_H_12_O_6_	180.0634	
Mannose	Man	C_6_H_12_O_6_	180.0634	
Hexose	Hex	C_6_H_12_O_6_	180.0634	
N-acetylgalactosamine	GaN	C_8_H_15_NO_6_	221.0899	
N-acetylglucosamine	GlN	C_8_H_15_NO_6_	221.0899	
N-acetylhexosamine	HeN	C_8_H_15_NO_6_	221.0899	
N-acetyl-neuraminic acid	NAN	C_11_H_19_NO_9_	309.1060	
N-glycolyl-neuraminic acid	NGN	C_11_H_19_NO_10_	325.1009	
2-AB	Tag		138.0793	
ProCA	Tag		237.1841	

#### Fragment generator

The Fragment Generator is another useful feature to add new glycans that are not in the current databases, expanding the range of glycan species that can be detected based on the specific needs of the analyst. The app’s algorithm generates the glycan structure as a MATLAB graph object with nodes representing the different monosaccharides and edges representing the bonds connecting them. B-, Y-, and internal ion fragments are generated by systematically cutting different bonds or monosaccharide connection points in the glycan and storing information about the resulting fragments, which are found by depth-first graph searching. The algorithm sequentially cuts one to seven bonds in each glycan to generate a full set of all mathematically possible glycan fragments, including internal ions resulting from multiple cuts. This feature helps prevent data processing search errors as the glycan fragmentation masses are automatically generated based on the glycan structure. A detailed description of how to write the structure code of a new glycan in a format that is accepted by the app is available in the GlyKAn AZ Instructions Manual (Additional file 1).

## Results

We collected LC–MS/MS data for various IgG and fusion protein glycan species and their GlyKAn AZ app results were compared to manually interpreted results to validate the outcomes. Evaluation results for one of these molecules is presented in the following sections.

### MS1 results

To demonstrate the capability and performance of the app, an example MS/MS data set for a monoclonal antibody was used for evaluation. An excerpt of the MS1 results directly from the app’s Excel output is shown in Table 4. The sample was generated according to the workflow described in the Implementation section. Glycans were labeled with procainamide as the fluorescent tag and were run using the GlyKAn AZ app with a maximum charge value of 2.

**Table 4:** Excerpt of MS1 output for example MS/MS data set

Name	Isomer peak no	RT (min)	Charge (z)	Observed m/z	Theoretical mass (Da)	Observed mass (Da)	Mass error (ppm)	Intensity (count)	Relative intensity (%)
A2G0M4F	1	13.393	2	922.9	1843.8	1843.8	5.5	7,590,580	100
A2G0M4F	2	13.778	2	922.9	1843.8	1843.8	5.2	1,614,040	100
A2G0M4F	3	14.079	2	922.9	1843.8	1843.8	1.1	36,308	32
A1G1F(α1,6)	1	13.393	2	922.9	1843.8	1843.8	5.5	7,590,580	100
A1G1F(α1,6)	2	13.778	2	922.9	1843.8	1843.8	5.2	1,614,040	100
A1G1F(α1,6)	3	14.079	2	922.9	1843.8	1843.8	1.1	36,308	32
A1G1F(α1,3)	1	13.393	2	922.9	1843.8	1843.8	5.5	7,590,580	100
A1G1F(α1,3)	2	13.778	2	922.9	1843.8	1843.8	5.2	1,614,040	100
A1G1F(α1,3)	3	14.079	2	922.9	1843.8	1843.8	1.1	36,308	32

As described in the Implementation section, the potential locations of isomer peaks are identified based on the intensity versus RT spectra for specific m/z ranges. Because A1G1F(α1,6), A1G1F(α1,3), and A2G0M4F have the same mass, three isomer peaks were identified at RTs of 13.39, 13.77, and 14.07 min. The MS1 output allows the analyst to briefly assess the likelihood that the isomer is present in the sample, particularly by looking at the intensity and relative intensity columns. Table 4 shows that isomer peaks 1 and 2, which occur at 13.39 and 13.77 min, respectively, are much more likely to correspond to glycan presence than is isomer peak 3, due to the higher relative intensities.

In this case, the RTs of glycan isomers were previously confirmed with glycan standards and/or MS/MS (data not shown). If relative RTs of isomers are unknown, then the MS2 tab might allow the analyst to identify which of those isomers belong to which RT, based on the fragmentation spectra and fragment masses that are unique to each isomer.

The MS/MS sample referenced above contained 20 glycans that were manually identified by analysts using Thermo Fisher Scientific XCalibur software [23]. Those results were compared with the MS1 results generated by the app for different mass accuracies and relative intensity thresholds. A proprietary database of approximate 170 glycans was used for MS1 peak identification. The true- and false-positive rates based on different set points of mass accuracy and relative intensity threshold are shown in Table 5. A relative threshold of 25% and mass error threshold of 10 parts per million (ppm) provided the optimal result for this dataset, containing the fewest false positives while still identifying all 20 confirmed glycans (including isomers). These false positives can be eliminated by using the MS2 peak confirmation functionality of the app or by considering absolute intensity. All 20 true positives were identified by the app at the same RTs as with manual analysis.

**Table 5:** MS1 performance for glycosylation sample

Relative intensity threshold (%)	Mass accuracy = 50 ppm		Mass accuracy = 10 ppm	
	True positive	False positive	True positive	False positive
0.20	20	144	20	44
5	20	51	20	30
10	20	29	20	18
25	20	13	20	12
50	19	8	19	7
100	16	6	16	6

### MS2 results

**Fig. 4:**
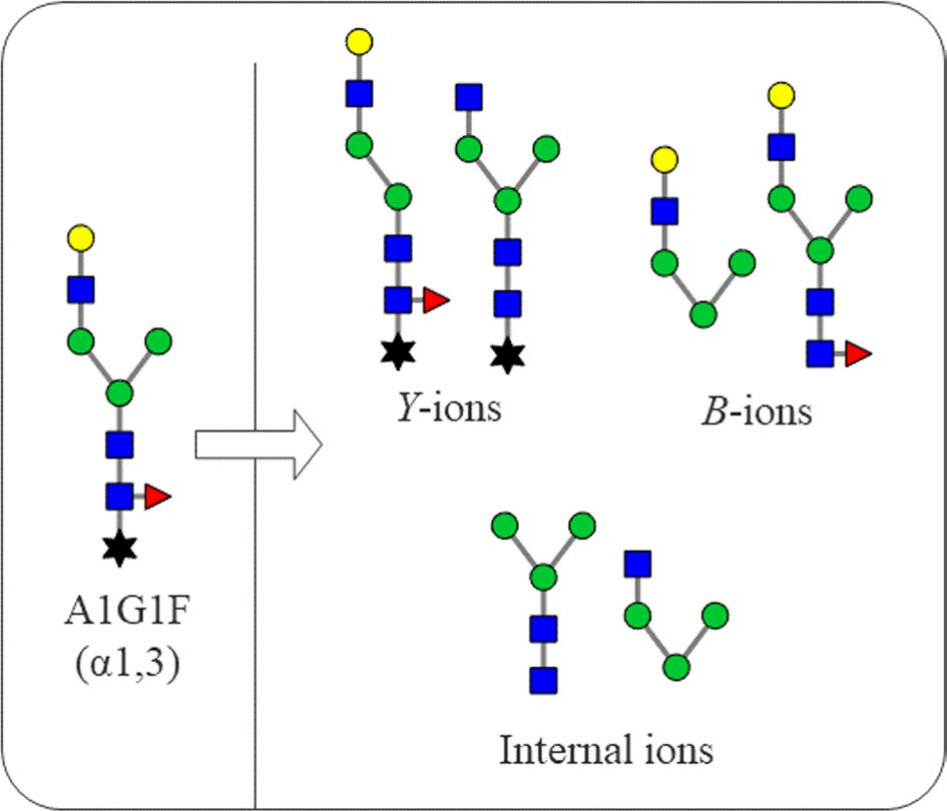
A1G1F(α1,3) potential fragments. Examples of some internal ions, B-ions, and Y-ions that would be auto-generated for glycan A1G1F(α1,3) via the GlyKAn AZ app’s fragment generator feature

**Fig. 5:**
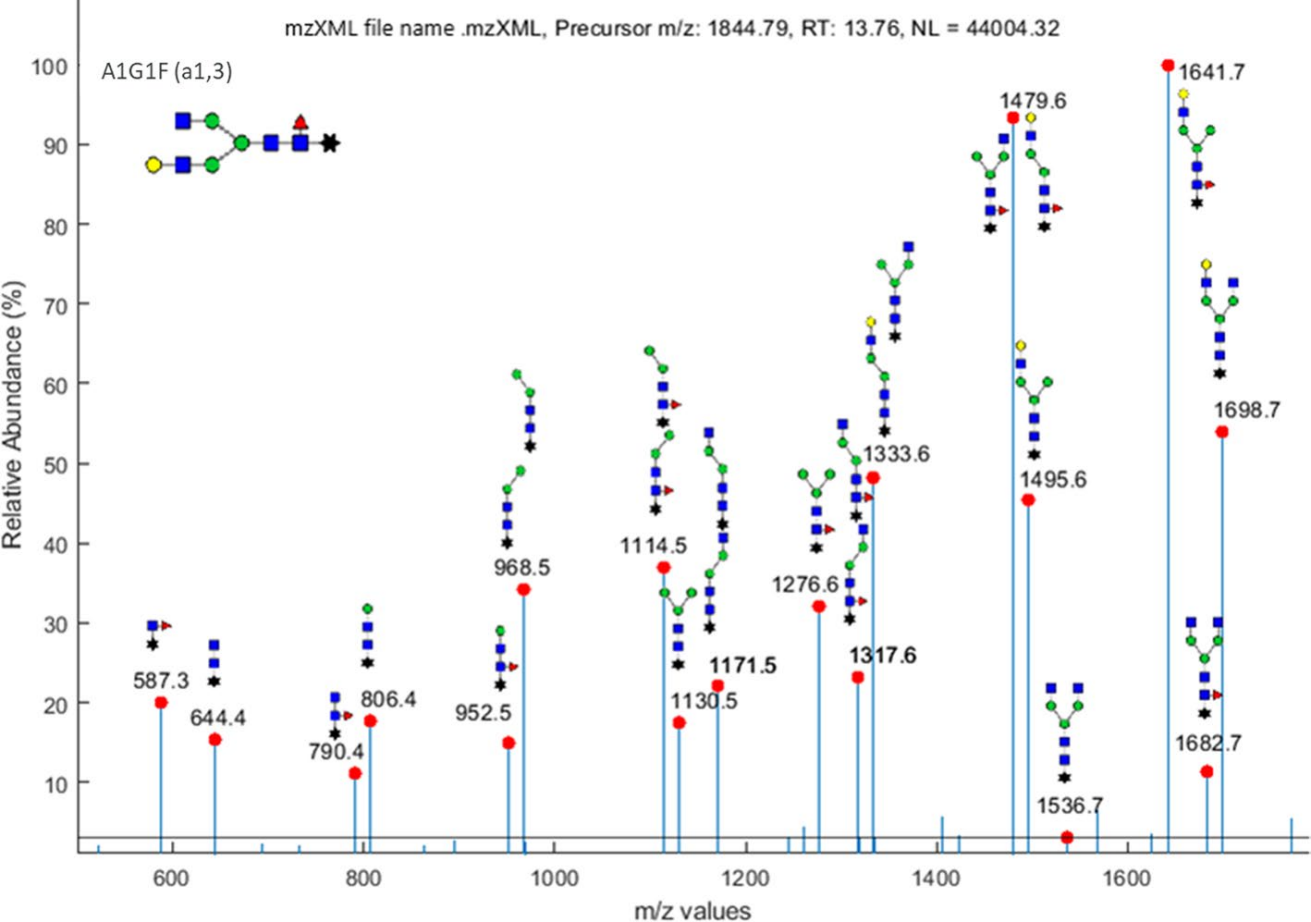
Example MS2 output for glycan A1G1F(α1,3). The spectrum shown is the annotated spectrum for glycan A1G1 (α1,3) auto-generated and labeled via the GlyKAn AZ app’s MS2 tab

Having identified potential glycan matches for the example MS/MS sample data via the MS1 tab, fragments can now be matched to the fragmentation spectra. When the glycan databases are created, potential fragment ions are permutated by the MS2 Fragment Generator tab, as discussed in the Implementation section. The resulting fragment images are saved and the fragment masses are added to the MS2 database. Figure 4 shows a few fragments created for glycan A1G1F(α1,3). These fragment images are used by the app to annotate the MS/MS fragmentation spectra. After the user selects one or several glycan-RT pairs to view, the app generates the annotated figures. Figure 5 shows the output of the MS2 Peak Confirmation tab for A1G1F(α1,3) at an RT of 13.77 min from the same data set presented in the preceding section. The figure shows that, of the 24 total distinct peaks falling above a relative abundance percentage of 3%, 18 fragment peaks were annotated. The app places glycan fragments near each peak and allows the user to move the fragments around to ensure readability. The figures are generated to require minimal user modification and can be directly imported into written reports. Many peaks display two potential fragment ions with the same mass. Meanwhile, for comparison, a semi-manual analysis performed independently and assisted by XCalibur software identified only 12 peaks matching fragment ions for A1G1F(α1,3) at an RT of 13.77 min. These results show that automating such a mathematically intensive process as calculating potential ion masses and matching them to m/z values makes the app results more thorough and reproducible than those obtained by an analyst working manually, as time is not a constraint.

The Details tab generated for A1G1F (α1,3) at an RT of 13.77 min indicates that 39% of all potential A1G1F(α1,3) fragments were identified; this number was specifically calculated to consider only those fragments with unique masses that are longer than one monosaccharide unit, providing an additional tool for the analyst to confirm the accuracy of the glycan match. In some cases, the same tab will also list fragments that are unique to the selected glycan over other glycans with the same mass; however, as the isomer A1G1F(α1,6) is very similar structurally to A1G1F(α1,3), there are no unique glycan fragments between them. In this case, an analyst would need to rely on their expert knowledge to differentiate between the glycans; for example, the analyst performing the semi-manual analysis had previous knowledge that A1G1F(α1,3) elutes later than A1G1F(α1,6) due to a difference in hydrophobicity, which led to identifying A1G1F(α1,6) as an earlier peak at an RT of 13.39 min. No adducts were identified in the first tab of the app, which is reasonable when one considers the high proportion of spectra peaks that matched with non-adduct fragments.

### MALDI–TOF results

A MALDI–TOF sample data set for permethylated N-glycans was analyzed and the results are summarized in Table 6. For this data set, the difference between theoretical and observed mass for each glycan was observed to be within 3 Da, so the acceptable mass error was defined in daltons instead of parts per million. At the time of manual analysis, 49 glycans were positively identified and 1 peak was not identified. Each test of the app at a different mass accuracy threshold was able to label the unidentified peak with the same glycan, demonstrating the app’s ability to recognize peaks that are missed during manual analysis. For each case, the false positives occurred when glycans were doubly assigned to a peak; in other words, the actual glycan match and a false glycan match with the same m/z were paired by the app to the same peak. Overall, the glycan app was shown to identify glycans with accuracy similar to that of an analyst in substantially less time, illustrating its applicability and reliability.

**Table 6:** MALDI performance for sample data set (*n* = 3)

Mass accuracy (Da)	True positives	Extra peaks identified	False positives
6	49	1	5
3	49	1	1
1	40	1	0

### Processing time

One of the benefits of the GlyKAn AZ app is the speed with which it can process the data. Reading the mzXML file is the most time-consuming portion of running the app and takes approximately 2–3 min, depending on the size of the file. For MS1 peak identification, the glycan identification takes an additional minute. For MS2 peak confirmation, the expected additional processing time is about 1 min to read database information and another 20 s for each glycan undergoing MS2 analysis. For the fragment generator, the addition of simple glycans could take about 1 min per glycan. For more complex glycans, the processing time can be several minutes. For MALDI–TOF, it took approximately 7–10 s for the app to finish the analysis of a database containing 109 permethylated glycans. These values are true to a standard laptop computer with an Intel Core i5 processor. The MS1 and MS2 analysis described in the above sections for 20 positive glycans (32 total glycans considered) took 15 min of active app run time. Depending on the user, reviewing the MS1 output file and the MS2 annotated spectra could take an additional hour of time. In comparison, a manual analysis where the analyst selects each peak to investigate potential glycan matches, aided by a peak visualization software such as XCalibur software, can take anywhere from several hours to several days to positively identify a full spectrum of glycans. This analysis time depends on the complexity of the data set, the analyst’s prior knowledge of the glycan spectra, and the extent of MS/MS fragment annotation desired.

## Conclusions

The GlyKAn AZ app improves manual analyst workflows by automating the input, analysis, and output process of characterization for N-linked glycans. It bypasses the manual steps of characterization and presents the data in auto-generated figures, tables, and relevant statistics that are easily interpretable by an MS analyst, facilitating decisions based on the provided outputs. The app itself is MATLAB based and has a user-friendly interface. Multiple tabs represent different functionalities, including MS1 peak identification, MS2 peak confirmation, MALDI peak identification, and fragment generation, allowing users to add new glycans to the databases by simply providing the structure. In the example MS/MS data set evaluation, all 20 manually identified glycans were successfully identified by the app as matches. The selections were confirmed by inspecting the annotated figures and relevant statistics generated by the MS2 Peak Confirmation tab. For the example MALDI data set, all 49 confirmed glycans were selected by the app, in addition to one peak that was previously unassigned during manual analysis. These results show that the app is accurate across MS/MS and MALDI functionalities.

Future improvements to the app include adding the ability to process multiple mzXML files simultaneously. Several more customizable fields can be added to the user interface, including the isomer peak retention time range, which is currently a fixed value that meets most use cases. The inclusion of a de novo approach could also enhance the app’s ability to identify glycans not already located in the database. In addition, the scope of the app could be expanded to consider O-linked glycans, which would enhance its relevance to different fields of research. This app saves analysts’ time and reduces the bottleneck of manually analyzing MS data. By designing around the end user, the GlyKAn AZ app has all the capabilities required by a glycan expert to perform rapid and reproducible glycan analysis, identifying RTs and isomers and ultimately generating figures that can support glycan identification for both development and regulatory applications.

## Supplementary Information

**Additional file 1**. GlyKAn AZ Instructions Manual.

## Data Availability

Project name: GlyKAn AZ app. Project home page: https://github.com/ZaylaSchaeffer/GlyKAn-AZ-application. Operating system: Platform independent. Programming language: MATLAB. Other requirements: MATLAB 2020b or higher. License: MIT License. Any restrictions to use by non-academics: MIT License. Data underlying the findings described in this manuscript may be obtained in accordance with AstraZeneca’s data sharing policy described at https://astrazenecagrouptrials.pharmacm.com/ST/Submission/Disclosure.

## Abbreviations

2-AB: 2-Aminobenzamide
CID: Collision-induced dissociation
Da: Daltons
DHB: 2,5-Dihydroxybenzoic acid
DMSO: Dimethylsulfoxide
HILIC: Hydrophilic interaction chromatography
LC: Liquid chromatography
ppm: Parts per million
ProCA: Procainamide
MALDI–TOF: Matrix-assisted laser desorption/ionization–time of flight
MS: Mass spectrometry
MS/MS: Tandem mass spectrometry
m/z: Mass-to-charge ratio
PNGase: Peptide-N-glycosidase A
RT: Retention time
Tris: Tris(hydroxymethyl)aminomethane
UPLC: Ultrahigh-performance liquid chromatography
