# Supplementary material for: A MATLAB-based app to improve LC–MS/MS data analysis for N-linked glycan peak identification
Source: BMC Bioinformatics. 2023 Jun 17;24:259. doi: 10.1186/s12859-023-05346-5 (PMC10276461; doi:10.1186/s12859-023-05346-5)
Supplement: Supplementary file 1 — Additional file 1. GlyKAn AZ Instructions Manual. [file 12859_2023_5346_MOESM1_ESM.docx]

**GlyKAn AZ Instructions Manual**

The following instruction manual contains step-by-step instructions on the following topics:

- Converting RAW files to mzXML files
- Installing GlyKAn AZ
- Using GlyKAn AZ’s MS1 Peak ID, MALDI-TOF Peak ID, MS2 Peak Confirmation, and Fragment Generator
- Interpreting and Adjusting MS2 Figures
- File Specifications
- Expected Errors
- Expected Processing Time

Contents

[Raw File Conversion 3](#_Toc66791837)

[MSConvert Installation 3](#_Toc66791838)

[Conversion to mzXML (Uncentroided) 4](#_Toc66791839)

[Conversion to mzXML (Centroided) 5](#_Toc66791840)

[Installing GlyKAn AZ 8](#_Toc66791841)

[Using GlyKAn AZ 9](#_Toc66791842)

[GlyKAn AZ: MS1 Peak ID 9](#_Toc66791843)

[GlyKAn AZ: MALDI-TOF Peak ID 12](#_Toc66791844)

[GlyKAn AZ: MS1 Figures 14](#_Toc66791845)

[GlyKAn AZ: MS2 Peak Confirmation 15](#_Toc66791846)

[GlyKAn AZ: Fragment Generator 18](#_Toc66791847)

[Fragment Generator Structure Excel File 19](#_Toc66791848)

[Interpreting and Adjusting MS2 Figures 21](#_Toc66791849)

[File Specifications 24](#_Toc66791850)

[Expected Errors 25](#_Toc66791851)

[Expected Processing Time 26](#_Toc66791852)

# Raw File Conversion

The process of converting .RAW files to mzXML has been divided into two phases: installing ProteoWizard, the conversion software, and using ProteoWizard for the actual conversion. The steps are as follows:

## MSConvert Installation

Note: Because of licensing constraints, the data conversion functionality of ProteoWizard is only available for [Microsoft .NET Framework 4.0](http://proteowizard.sourceforge.net/download.html?id=17851) or higher installed.

1. Visit [http://proteowizard.sourceforge.net/download.html](mailto:amashaan@mit.edu) for ProteoWizard’s download page.
2. Select “ProteoWizard” for the project and “Windows 64-bit installer” as the platform.


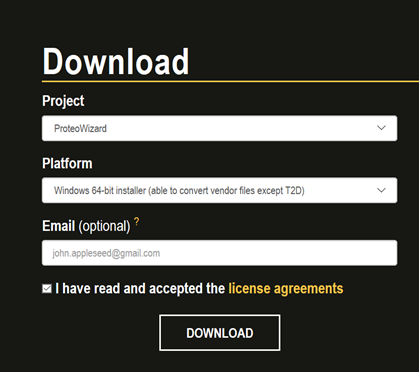


1. After the download is complete, open the installation file. Select “Install Just for you” as the Installation Scope.


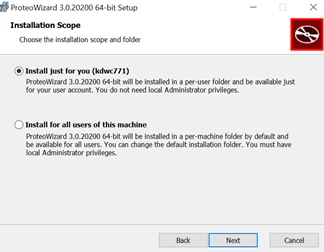


1. Select Both options “Add MSConverGUI to the Windows Explorer right click menu” and “Add SeeMS to the Windows Explorer right-click menu” to install the necessary files.


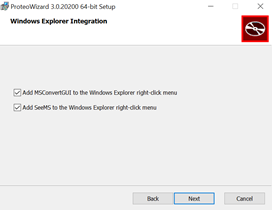


## Conversion to mzXML (Uncentroided)

1. To open the conversion software, search for “MSConvert” in the start menu. MSConvert is the name of the GUI that ProteoWizard uses.
2. To input the .RAW file, select “List of Files” and click on the “Browse” button to select the data file to be converted. Click on “Add” to add the file to the conversion list.
3. To determine the save location of the mzXML file, click on “Browse” under the “Output Directory.” The folder selected will be the location in which the mzXML file will be exported to.
4. To correctly convert the .RAW file to a type that MATLAB can read, select the following options from the “Options” section:
   - Output Format: mzXML (not mzML!)
   - Binary Encoding Precision: 32-bit
   - Write Index: on
   - Use zlib Compression: off
   - TTP Compatibility: on
   - Package in gzip: off
5. Click on “Start” in the bottom right corner to convert the .RAW file to mzXML format.


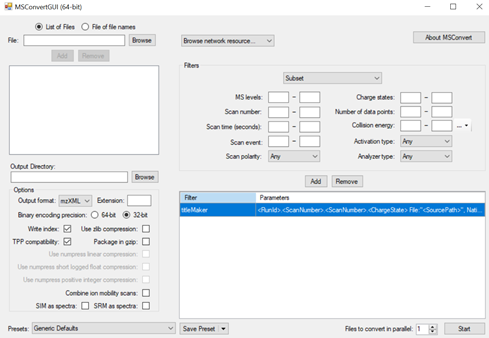


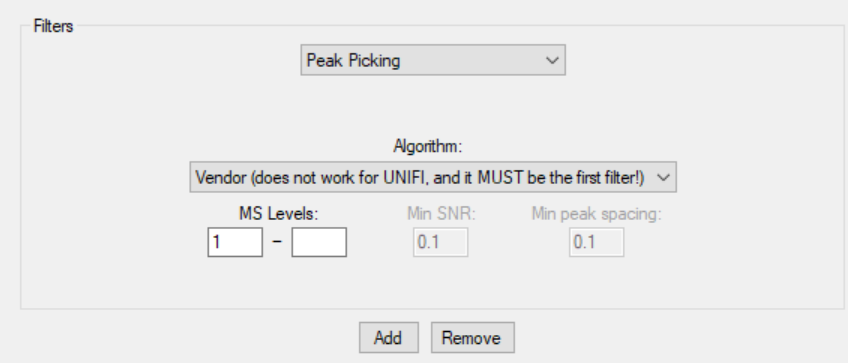


# Installing GlyKAn AZ

1. Double click the GlyKAn AZ MATLAB app installation file.


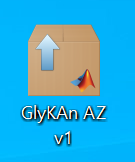


1. Click Install button to install the GlyKAn AZ app directly into MATLAB.


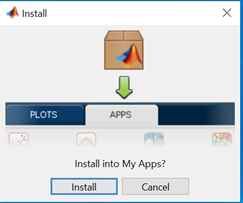


1. To open GlyKAn AZ, open MATLAB first. Under the “APPS” tab, GlyKAn AZ can be found in the “My Apps” section. For convenience, the app can be favorited (by clicking the star in the upper right corner) and dragged to the top of the list. Click on GlyKAn AZ to open it.


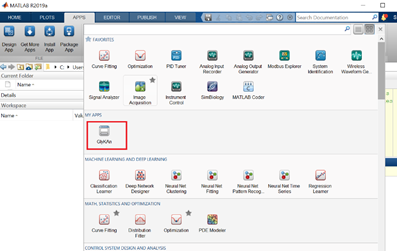


# Using GlyKAn AZ

## GlyKAn AZ: MS1 Peak ID

1. To input the uncentroided mzXML file, click on the “Browse” button next to “Select a mzXML File” to select the data file to be analyzed. The directory of mzXML file will be shown automatically.


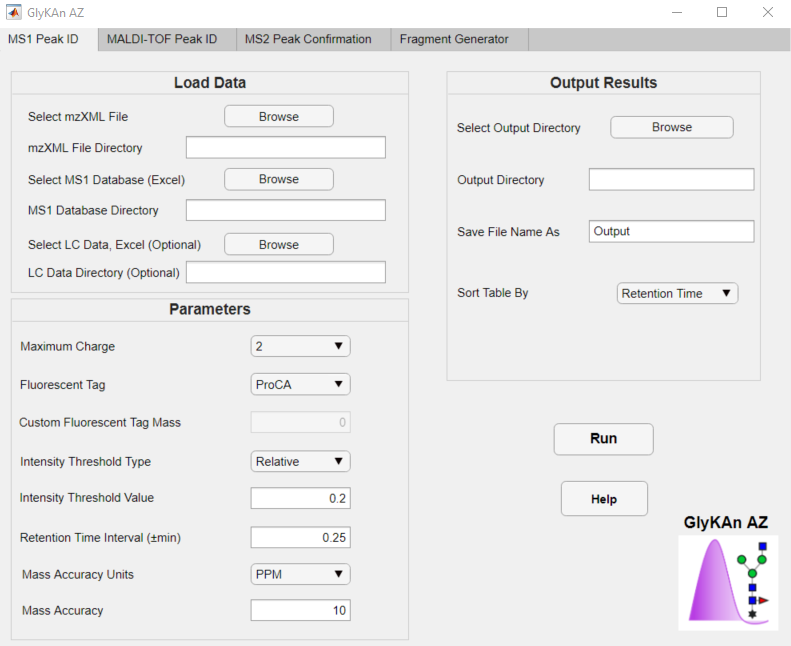


1. To input the MS1 database file, click on the “Browse” button next to “Select MS1 Database.” The database directory will be shown automatically.


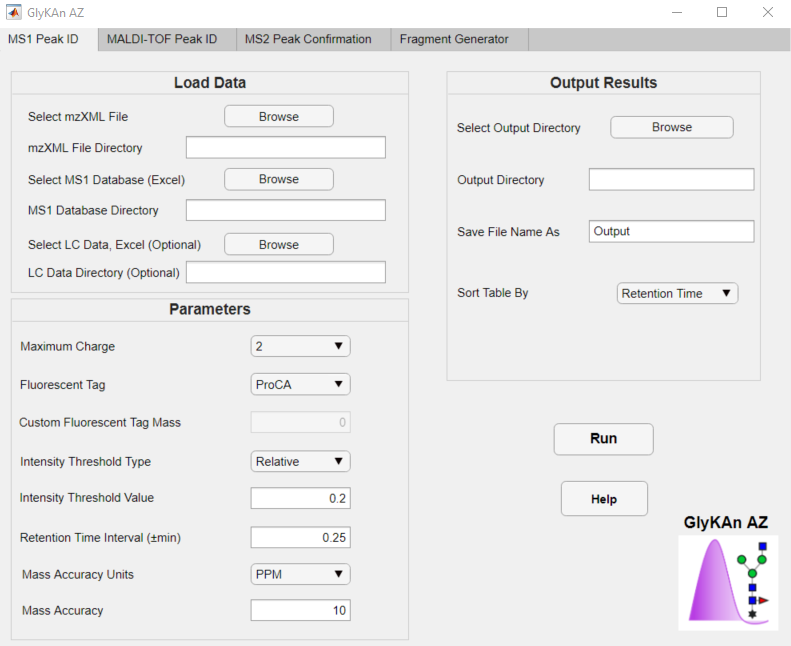


1. To input the Liquid Chromatography (LC) data, click on the “Browse” button next to “Select LC Data.” The database directory will be shown automatically. This input is optional and will help reduce the number of false positives by only searching relevant retention times.
   1. To generate LC data in a format understandable by GlyKAn AZ, import the raw LC file into MassLynx by clicking on “Chromatogram”  “Open”. Select the file you want to open and click OK.


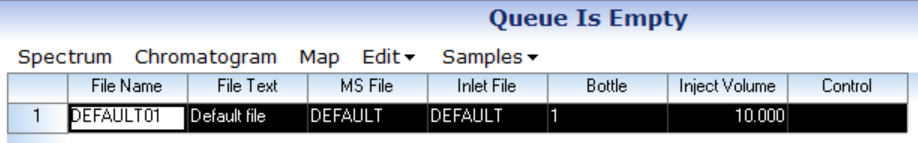


- 1. Once you have the trace you want, press the icon with two peaks (one green, one blue), and you will see the areas under the peak.


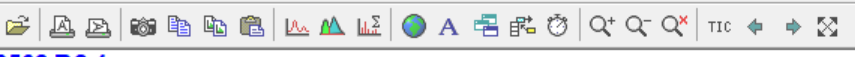


- 1. Click “Edit”  “Copy Detected Peaks” or select the icon with two pieces of paper. Paste this into cell **A1** of a blank Excel file. There should be 7 columns. This is the file you can upload to GlyKAn AZ.

1. Next, the tunable parameters for the analysis can be specified.
   1. Maximum Charge: For the analysis, the charge range must be specified for the code to convert mass to m/z for the scan. The value selected here is the maximum charge value that will be scanned for (for example, a selection of 3 means that m/z values corresponding to z = 1, 2, and 3 will be scanned).
   2. Fluorescent Tag: The tag used must be specified for the mass list to be updated. The code already contains information on 2-AB and ProCA, but can also use a custom tag.
   3. Custom Fluorescent Tag: If “Custom” is selected in the Fluorescent Tag field, the code will use the value inputted in this field. If the database used already incorporates the mass tag, a selection of “Custom” and a mass of 0 can be used.
   4. Intensity Threshold Type: Any peak with an intensity below a threshold value will be considered noise and not be scanned for the analysis. This field selects the type of intensity to be set for that threshold.
   5. Intensity Threshold Value: The value of the intensity threshold is then selected. If “Relative” is picked earlier, this value is a percentage. If “Absolute” is picked earlier, this value must be 0 or greater.
   6. Retention Time Interval: If a retention time is specified for a glycan in the MS1 database, the code will scan around that retention time. The retention time interval field specifies how far a search the code will scan, with the retention time provided corresponding to the center of the peak.
   7. Mass Accuracy Units: You can choose whether to specify mass accuracy units in Daltons (Da) or PPM.
   8. Mass Accuracy: Any peak with a mass error above this tolerance will not be considered a match.
2. In order to select location of the results table, click on the “Browse” button next to “Select Output Directory”. The output directory will be shown automatically.
3. To specify the name of results table, please enter the name to “Save File Name As.”
4. The data tables to be generated can be sorted by picking the “Sort Table By” field. The tables can be sorted by retention time (increasing), charge (increasing), m/z value (increasing) observed mass (increasing), mass accuracy (decreasing, absolute value), intensity (decreasing), and relative intensity (decreasing).
5. Click “Run ” button to start the analysis.

- If the user has any questions related to this section during the analysis, please click the “Help” button.

## GlyKAn AZ: MALDI-TOF Peak ID


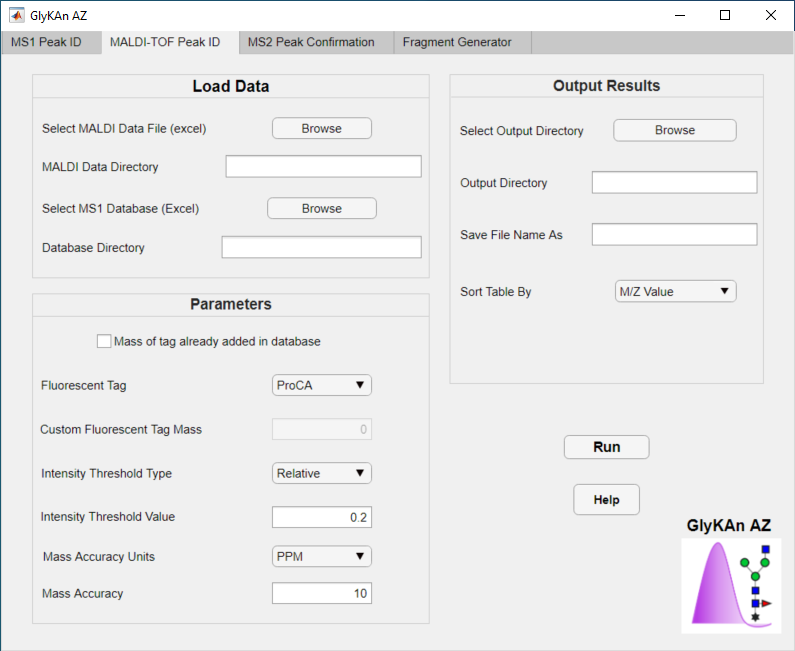


1. To input the MALDI instrument Excel file, click on the “Browse” button next to “Select MALDI Data File (excel)” to select the data file to be analyzed. The directory of the file will be shown automatically.
2. To input the database file, click on the “Browse” button next to “Select Database.” The database directory will be shown automatically.
3. Next, the tunable parameters for the analysis can be specified.
   1. Tag Mass Checkbox: If the mass of the tag and any adducts are already included in the masses in the database, check this box.
   2. Fluorescent Tag: If the tag mass is not included, then the tag used must be specified for the mass list to be updated. The code already contains information on 2-AB and ProCA, but can also use a custom tag.
   3. Custom Fluorescent Tag: If “Custom” is selected in the Fluorescent Tag field, the code will use the value inputted in this field. If the database used already incorporates the mass tag, a selection of “Custom” and a mass of 0 can be used.
   4. Intensity Threshold Type: Any peak with an intensity below a threshold value will be considered noise and not be scanned for the analysis. This field selects the type of intensity to be set for that threshold.
   5. Intensity Threshold Value: The value of the intensity threshold is then selected. If “Relative” is picked earlier, this value is a percentage. If “Absolute” is picked earlier, this value must be 0 or greater.
   6. Mass Accuracy Units: You can choose whether to specify mass accuracy units in Daltons (Da) or PPM.
   7. Mass Accuracy: Any peak with a mass error above this tolerance will not be considered a match. You may select these either using units for PPM or Daltons (Da) as specified above.
4. In order to select location of the results table, click on the “Browse” button next to “Select Output Directory”. The output directory will be shown automatically.
5. To specify the name of results table, please enter the name to “Save File Name As.”
6. The data tables to be generated can be sorted by picking the “Sort Table By” field. The tables can be sorted by m/z value (increasing), observed mass (increasing), mass accuracy (decreasing, absolute value), intensity (decreasing).
7. Click “Run ” button to start the analysis.

## GlyKAn AZ: MS2 Peak Confirmation


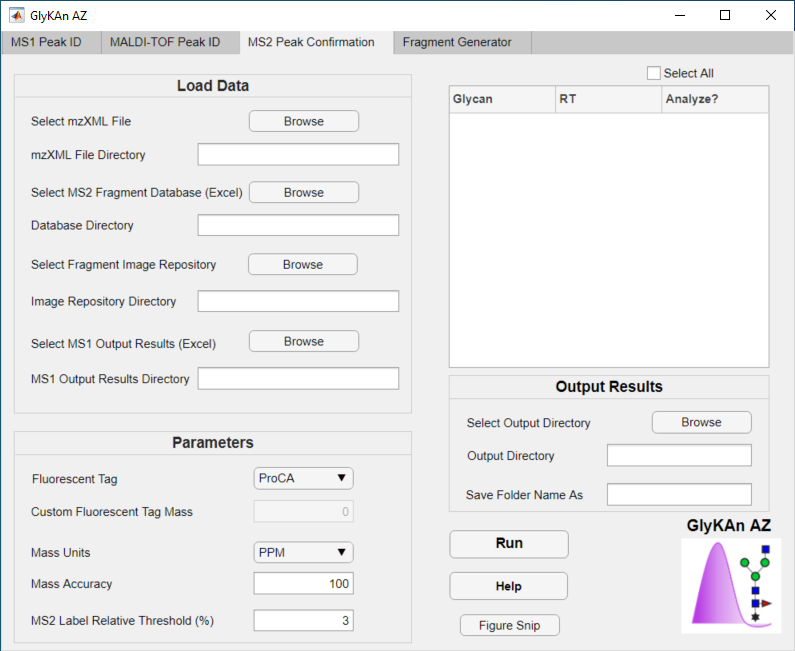


1. To input the uncentroided mzXML file, click on the “Browse” button next to “Select a mzXML File” to select the data file to be analyzed. The directory of mzXML file will be shown automatically.
2. To input the database file, click on the “Browse” button next to “Select MS2 Fragment Database (Excel).” The database directory will be shown automatically.
3. To input the image repository folder, click on the “Browse” button next to “Select Fragment Image Repository”. The image directory will be shown automatically. Keep in mind that this is the main folder that contains all of the subfolders for each glycan.
4. To input the MS1 output file, click on the “Browse” button next to “Select MS1 Output Results (Excel)”. This directory will be shown automatically. Keep in mind that this is the unchanged MS1 results file.
5. Once you complete step 4, a table will appear on the right hand side of the GUI with a list of all possible glycans to analyze from the MS1 output result file. Some glycans will be listed in several lines with different retention times, corresponding to potential isomer peaks. Click on the glycan & retention time pairs you want to perform an MS2 analysis of. Alternatively, check “Select all” for all of them.
6. Next, the tunable parameters for the analysis can be specified.
   1. Fluorescent Tag: The tag used must be specified for the mass list to be updated. The code already contains information on 2-AB and ProCA, but can also use a custom tag.
   2. Custom Fluorescent Tag: If “Custom” is selected in the Fluorescent Tag field, the code will use the value inputted in this field. If the database used already incorporates the mass tag, a selection of “Custom” and a mass of 0 can be used.
   3. Mass Accuracy Units: You can choose whether to specify mass accuracy units in Daltons (Da) or PPM.
   4. Mass Accuracy: Any peak with a mass error above this tolerance will not be considered a match. You may select these either using units for PPM or Daltons (Da) as specified above.
   5. Relative Intensity Threshold: Any peak with a relative intensity (%) below this value will be considered noise.
7. In order to select location of the results table, click on the “Browse” button next to “Select Output Directory”. The output directory will be shown automatically.
8. To specify the name of the folder in which MS2 results will be stored in, please enter the name to “Save Folder Name As.”
9. At this point, click “Run” to obtain figures. If the code cannot find an MS2 spectrum for a particular selected glycan and retention time, it will output a message in a pop up message box. The annotated figures that it does find will be placed in a created folder in the specified directory.
10. You may reopen the figures as MATLAB figure files. The “Figure Snip” button on the GUI allows you to crop to the edges of the figure (without the top tabs showing) as well as save as a png. In order to use this, open the figure in MATLAB, go to the desired tab, and click “Figure Snip”. The new png figure will be saved with the same name (along with an underscore and the name of the tab) to the original file location.

## GlyKAn AZ: Fragment Generator


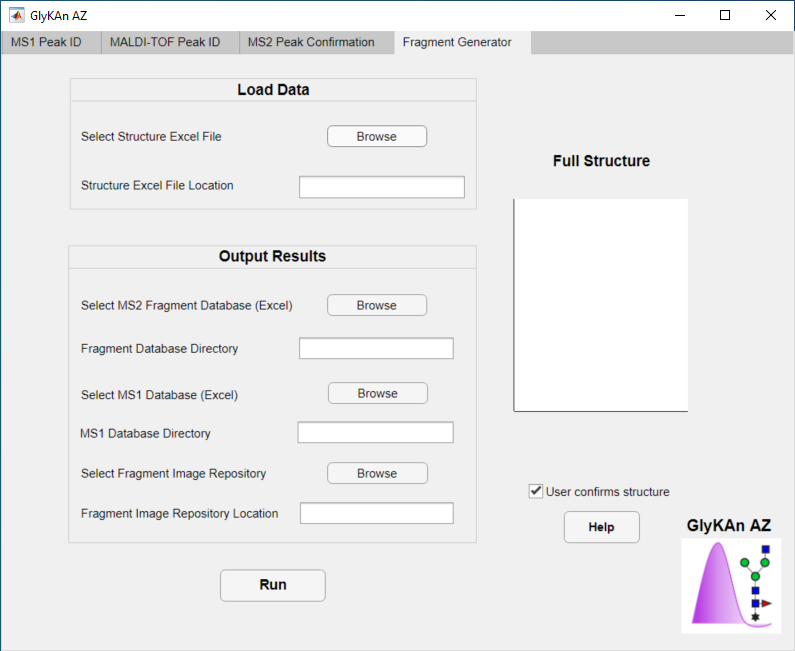


1. To access the Fragment Generator, open the GlyKAn AZ app and select the “Fragment Generator” tab.
2. To input the structure excel file, click the “Browse” button next to “Select Structure Excel File”. The contents of the structure excel file will be described at the end of this section.
3. Select the MS2 Fragment Database you would like the fragments to be added to by clicking the “Browse” button next to “Select MS2 Fragment Database (Excel)”. The fragment details will be added at the bottom of the sheet.
4. Select the MS1 Database you would like the glycan(s) to be added to by clicking the “Browse” button next to “Select MS1 Database (Excel)”. The glycans will be added at the bottom of the sheet.
5. Select the Fragment Image Repository you would like the fragment images to be added to by clicking the “Browse” button next to “Select Fragment Image Repository”. The fragments will be added in new folders for their respective glycans.
6. Please leave the “user confirms glycan” box checked unless you are regenerating the entire database in which case the GUI will not ask you to confirm the structure of each glycan.

### Fragment Generator Structure Excel File

The fragment generator structure excel file is an excel sheet that contains the glycan’s name, mass, and a section of structure code that details how the sugar units in the glycan are connected. Each cell describes a different sugar, starting from the Tag (if your glycan has a Tag). The first number describes what the sugar (or tag) is attached to; the next few spaces are a 3-letter code describing what type of sugar it is, and the second number labels that specific sugar. For example, the tag at the start of the glycan would be labelled "0 Tag1", because it is the first item in the glycan and is therefore not connected to anything upstream (hence the “0”). This tag is unit 1. The second cell could be "1 GlN2", describing a GlcNAc attached to unit 1 (the Tag) which is numbered as unit 2. An example is shown below for A1G0F. Each sugar unit needs its own individual number. Some special cases are described below the example.

The columns on the structure excel file must be labelled “Common Name”, “Mass” or “MW”, “Bisected”, and “Structure Code”. The GUI will search for those key words and use them to assign meaning to each column. It is ok to have empty columns in between them. “Structure Code” should be written over the first column containing the code (usually “0 Tag 1”).


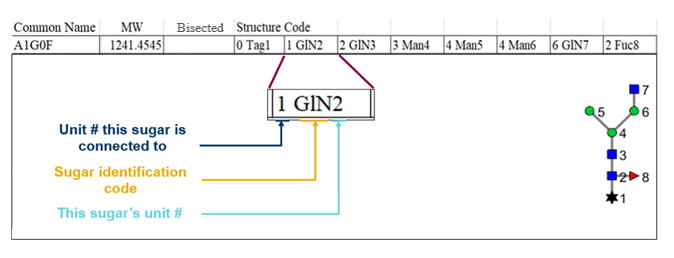


The total sugar identification codes are:

- Tag = the tag (usually ProCA or 2-AB)
- GlN = GlcNAc
- GaN = GalNAc
- HeN = HexNAc (generic)
- Gal = Galactose
- Man = Mannose
- Hex = Hexose (generic)
- Fuc = Fucose
- NAN = Neu5Ac
- NGN = Neu5Gc
- Xyl = Xylose

#### Isomers

This is for the case where there are structurally-significant isomers for a single glycan name. In the excel input file, the name should be followed by an isomer identifier, such as “(Isom 1)”, “(a)”, or information about the structural uniqueness of the glycan (such as “(a1,3)”). This will help distinguish the isomers in the database. For the structure, the code will assign positions in descending order from left to right. At a branching point, if you want a particular branch to appear on the left, its first sugar must be labelled a lower number than the first sugar of the branch on the right.

*Examples of structurally-significant isomers for A3S1G0F*

*
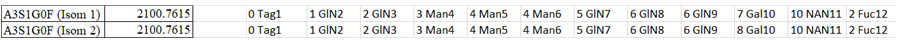
*


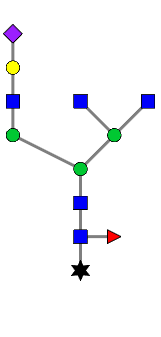

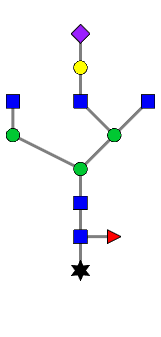


#### Bisected glycans

If you have a bisected glycan, you will need to define the structure very carefully. For the example of a mannose branching off into two mannose and one GlcNAc, where the GlcNAc is bisecting, the GlcNAc needs to be numbered between the other two mannoses. The left mannose would be 4 Man5, the GlcNAc would be 4 GlN6, and the right mannose would be 4 Man7. In the input excel file, there must be a column named “Bisected”, and bisected glycans need an “x” marked in that column which will signal to the code to straighten the bisecting portion.

*Example of Bisected glycan: G2F+NGNA2+GN*

*
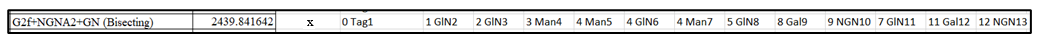
*


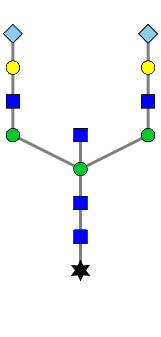


# Interpreting and Adjusting MS2 Figures

1. In the image folder, there will be 2 file types for each figure: “MATLAB Figure” and “MATLAB Code”. Click on the “MATLAB Figure” file type to open the figure.
2. The MS2 image outputs contain 3 tabs: Main, Cartoons, and Details.
   1. Main tab: Contains fragment peak identification including adducts
   2. Cartoons tab: Contains fragment peak identification and their corresponding cartoons located above the peaks. Up to 2 potential fragments for each detected peak will appear. These will either be different fragments with the same m/z, distinguishable isomers, or indistinguishable isomers. In the case of indistinguishable isomers, one of them can be deleted.
   3. Details tab:
      1. Contains unique mass information. If the display says “unique full mass”, then that means the total mass of the parent glycan is not present anywhere else in the database. If the display says “unique fragments found:…”, then the subsequent masses are unique masses for that specific charge that are not found elsewhere in the database among other glycans.
      2. Contains percentage information. The percent displayed here is the percentage of algorithm-identified possible masses that were identified as peaks in the spectrum. The higher the percentage, the more glycan fragments the spectrum captures. Therefore this number can be loosely correlated with the level of confidence that the glycan is present in the sample.
3. MATLAB figures are very editable. To edit cartoons or text, start by selecting the “Edit Tool” cursor at the top.


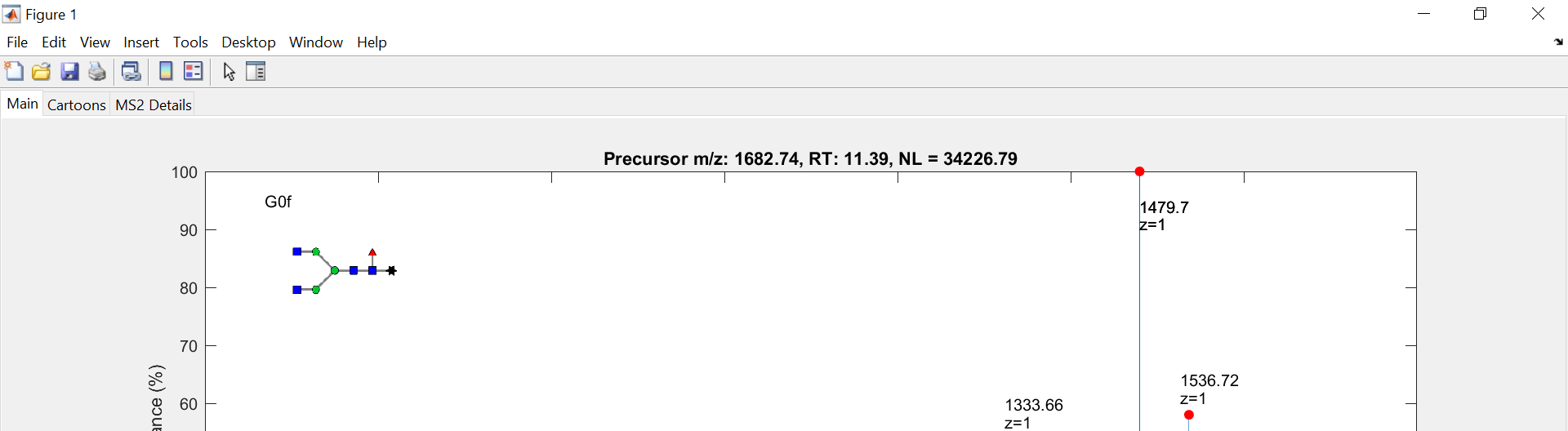


1. To edit the text annotations: Select the text box. Once the selection is made, you can drag, delete, resize, or even edit the actual text with your cursor.


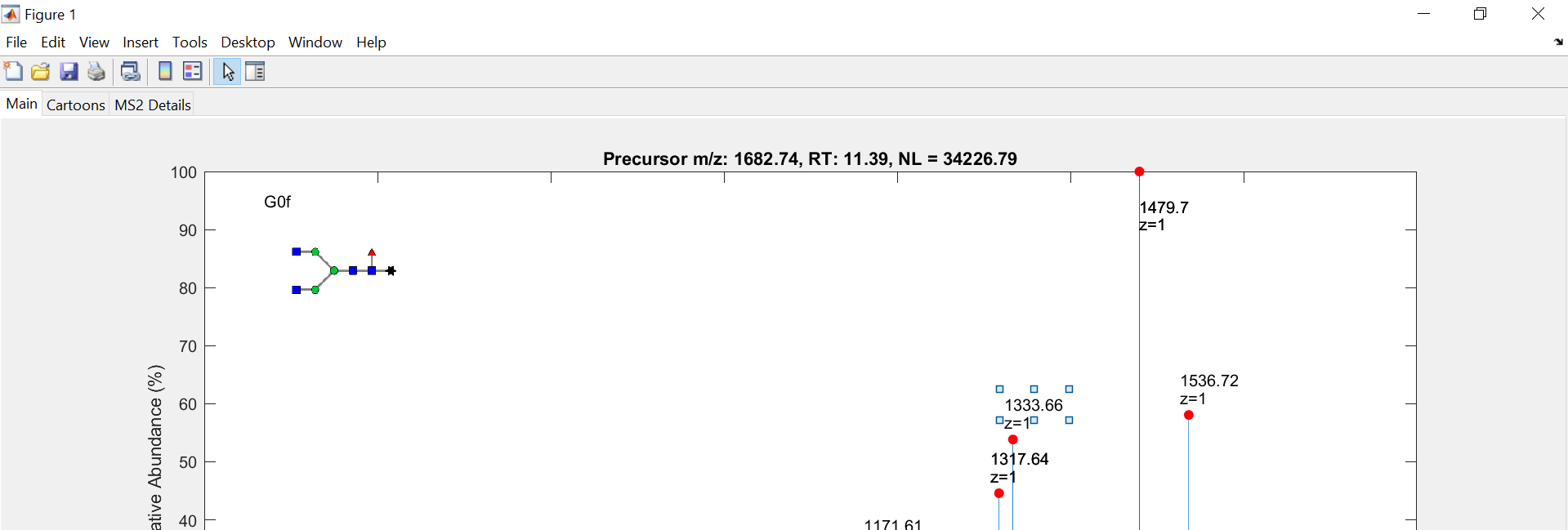


1. To drag, resize, or delete the cartoons:
   1. Double-click the cartoon. The “Property Inspector” window should pop up. If you want to delete the cartoon or resize, you can do that at this point.


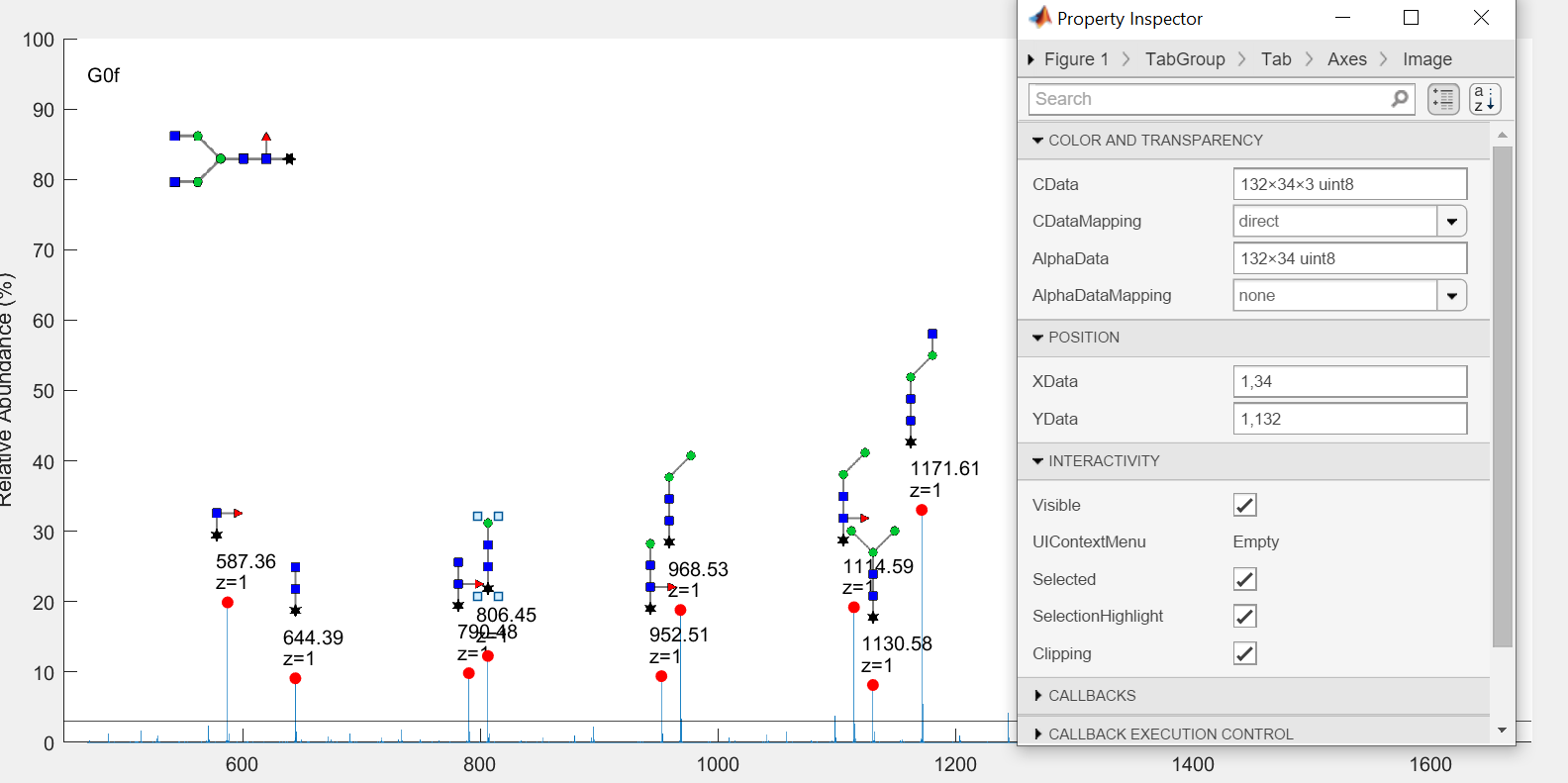


- 1. Click “Axes” at the top of property inspector, and the property inspector should reroute the path to stop at “Axes.”


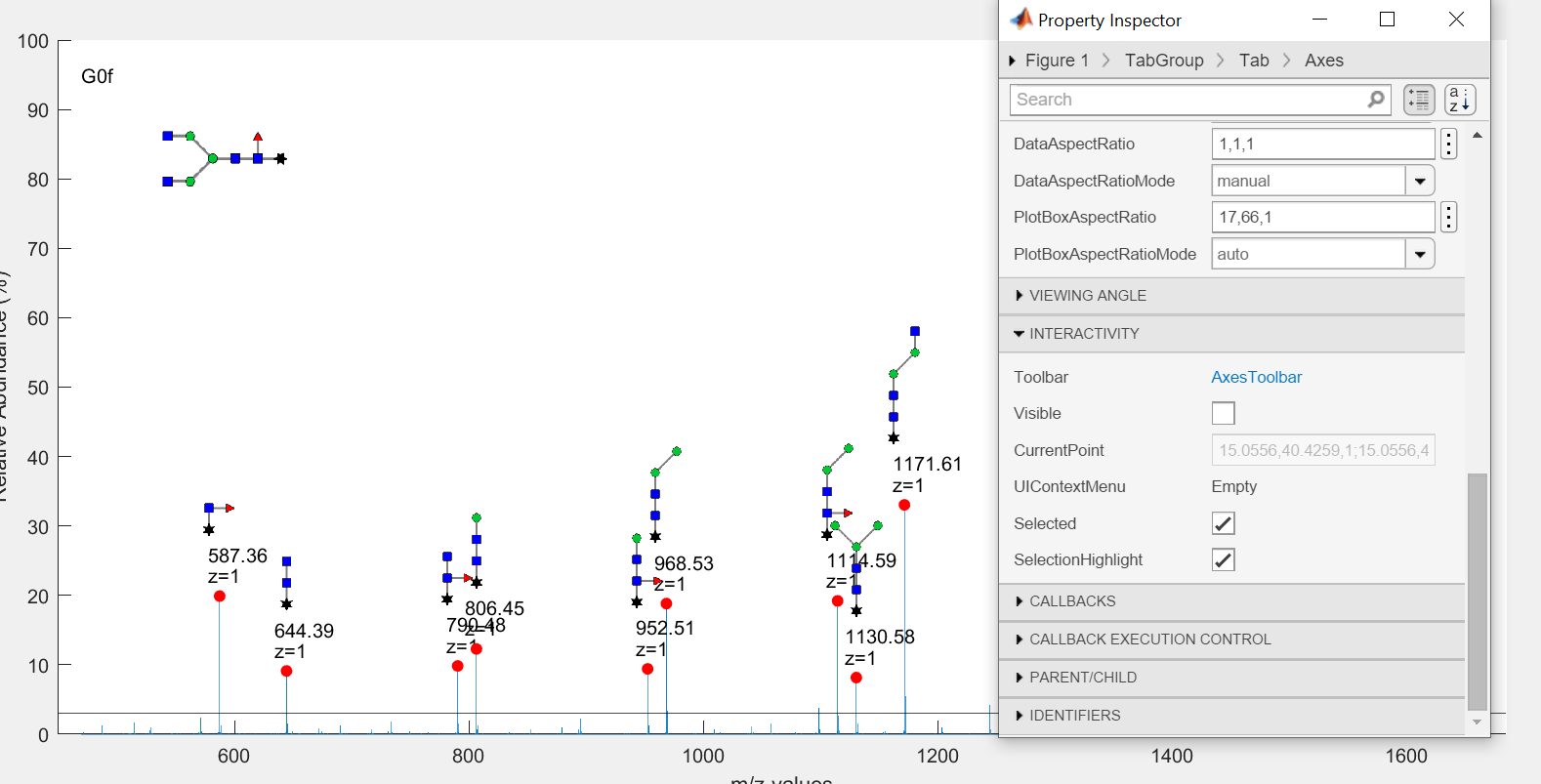


- 1. Check the “Visible” box under “Interactivity” and the axes should appear for the cartoon. At this point, you can drag the cartoon wherever you want using the 4-way scroller cursor.


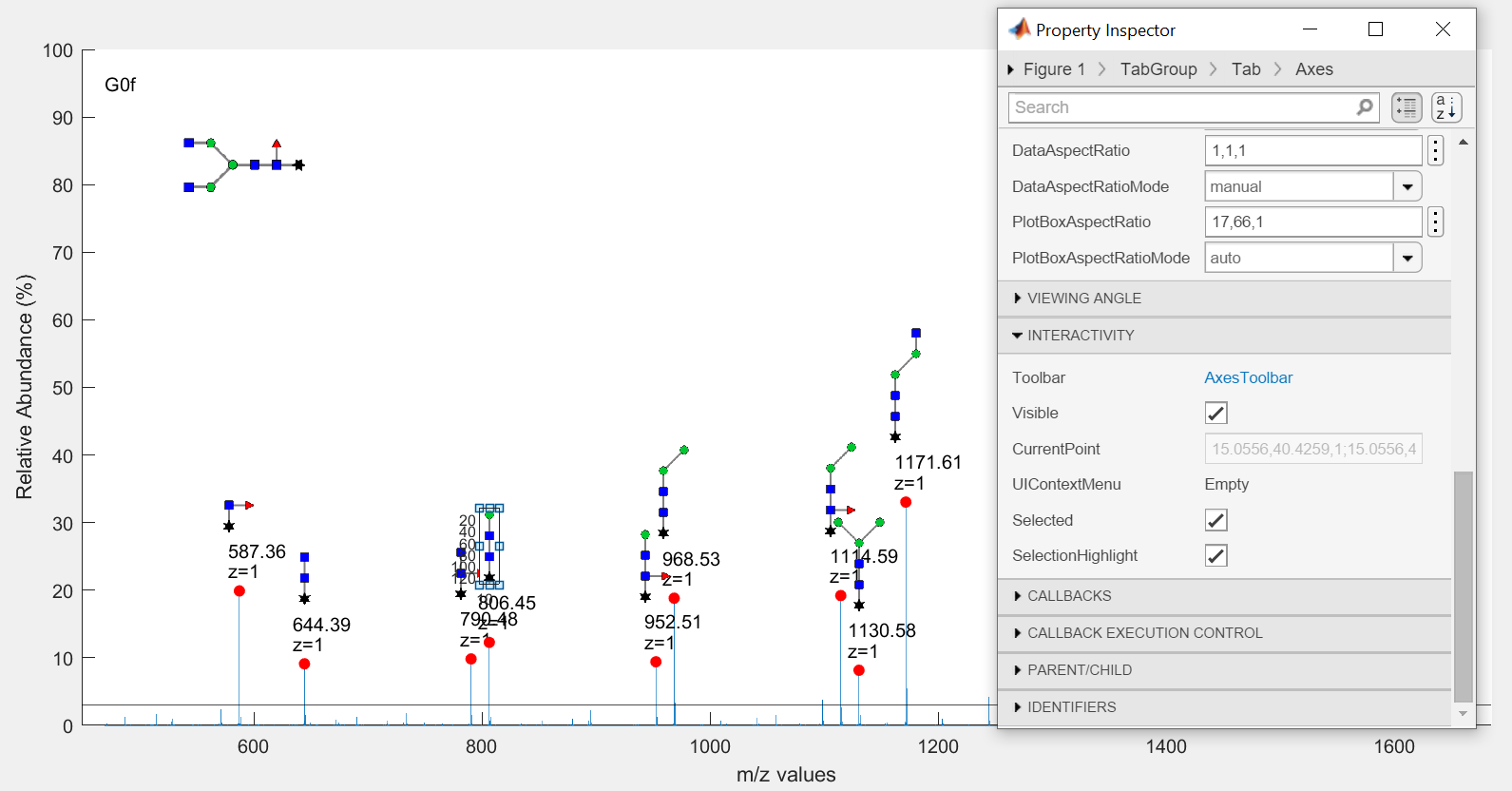


- 1. Once you’re done, uncheck the “Visible” box and either close Property Inspector or choose another cartoon.


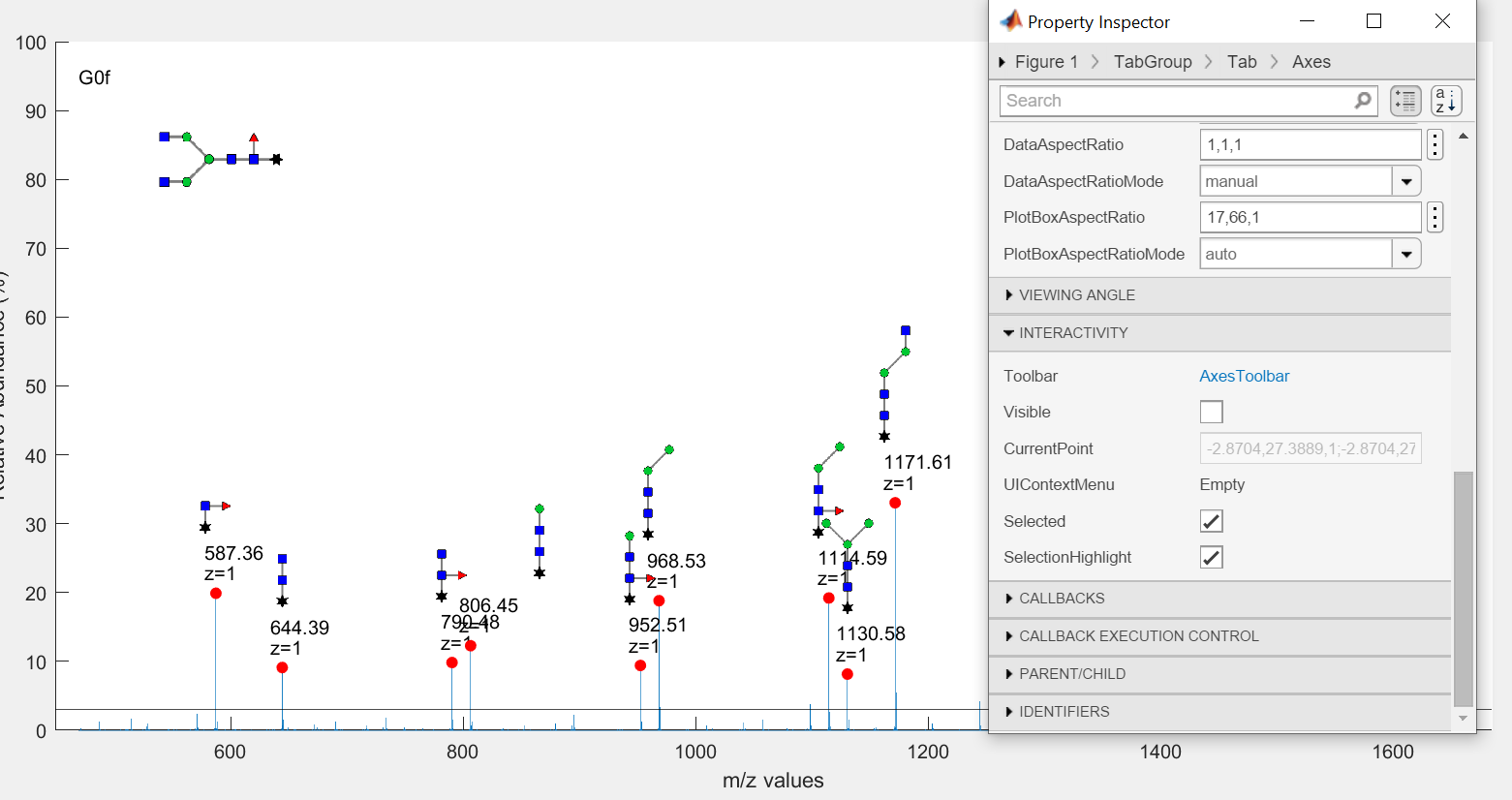


1. Once all annotation edits are completed, click the “Edit Tool” cursor again to exit edit mode. You can now crop and save the cartoon tab using the “Figure Snip” button on the GUI.

# File Specifications

1. For MS1 Peak ID, the database table must contain the following headers: Common Name, Mass (Da), Retention Time (min). The code checks the title of the first column to make sure the correct data file is selected. If the wrong file is selected, an error message will be displayed. The retention time for each glycan is optional, but the column name must still be present. All other columns must be completely filled. If those values are not specified, an error message will be displayed. The mzXML file must be uncentroided.
2. For MALDI-TOF Peak ID, the raw data file must be the Excel file outputted by the MALDI instrument. The database table must contain the following headers: Common Name and Mass.
3. For MS2 Peak Confirmation, the fragment database and image repository must be of the same version that was used to run MS1. For example if new glycans were added to the MS1 database without going through the “Fragment Generator” tab, then the MS2 database will not contain those new glycans and their fragments. The code will throw errors if someone attempts to run those glycans on MS2. The mzXML file must uncentroided.
4. The Fragment Generator Structure Excel File is described in the GlyKAn AZ: Fragment Generator section.

# Expected Errors

1. If any inputs are left blank, an error message stating “Please Select a File” will be displayed.
2. If incorrect database is inputted, an error message stating “Incorrect data file. Please consult final report for Mass List file specifications” will be displayed.
3. If incorrect mzXML is inputted, an error message stating “Incorrect mzXML file. Please consult the mzXML file conversion instructions for more information” will be displayed.
4. If the LC peaks excel file is not in the expected format, an error message stating “LC data not found in correct columns, please refer to manual” will be displayed.
5. Other errors can occur if the databases or image repository are in the incorrect format. Keep in mind that the code relies on correct column names and order, sheet names and order, glycan names in the MS2 database exactly matching those in the image repository, and order of fragments in rows in the MS2 database. Avoid making any of the aforementioned changes to the databases.
6. Errors can occur on the MS2 tab if different versions of the MS1 and MSMS database are being used. The code relies on being able to match names exactly. The original versions of both these databases were provided, and they are updated in tandem when new glycans are added using the “Fragment Generator” tab.
7. Errors can also occur in the MS2 tab if the glycan name is not unique.

# Expected Processing Time

1. MS1 Peak ID: The expected processing time is 2 to 3 minutes. If more than 5 minutes has elapsed, please look at MATLAB’s Command Window to check if any errors are displayed. Some mzXML files are much larger than others, so it’s possible that MATLAB is just taking a long time to read it. For files over 1 GB, we recommend using a high-processing power computer.
2. MALDI-TOF Peak ID: The expected processing time is on the order of several seconds. This could range from about 20-30 seconds depending on the size of the database and raw files.
3. MS2 Peak Confirmation: The expected processing time depends on the number of glycans selected to analyze. You can expect about 1 to 3 minutes to read the mzXML file, about 1 minute to read database information, and another 20 seconds for each glycan you want to analyze.
4. Fragment Generator: For simple glycans, the processing time will be ~1 minute per glycan. For complicated glycans, the processing time can be several minutes. User inputs are required for each glycan during the run (to confirm the structures) so be prepared to wait for the process to complete.
